# Vibroacoustic Response of the Tympanic Membrane to Hyoid-Borne Sound Generated during Echolocation in Bats

**DOI:** 10.1093/iob/obad004

**Published:** 2023-01-28

**Authors:** C C G Snipes, R T Carter

**Affiliations:** Department of Biological Sciences, East Tennessee State University, 1276 Gilbreath Dr, Johnson City, TN 37614, USA; Department of Biological Sciences, East Tennessee State University, 1276 Gilbreath Dr, Johnson City, TN 37614, USA

## Abstract

The hyoid apparatus in laryngeally echolocating bats is unique as it forms a mechanical connection between the larynx and auditory bullae, which has been hypothesized to transfer the outgoing echolocation call to the middle ear during call emission. Previous finite element modeling (FEM) found that hyoid-borne sound can reach the bulla at an amplitude likely heard by echolocating bats; however, that study did not model how or if the signal could reach the inner ear (or cochlea). One route that sound could take is via stimulation of the eardrum—similarly to that of air-conducted sound. We used micro computed tomography (μCT) data to build models of the hyoid apparatus and middle ear from six species of bats with variable morphology. Using FEM, we ran harmonic response analyses to measure the vibroacoustic response of the tympanic membrane due to hyoid-borne sound generated during echolocation and found that hyoid-borne sound in all six species stimulated the eardrum within a range likely heard by bats. Although there was variation in the efficiency between models, there are no obvious morphological patterns to account for it. This suggests that hyoid morphology in laryngeal echolocators is likely driven by other associated functions.

## Introduction

**Fig. 1:**
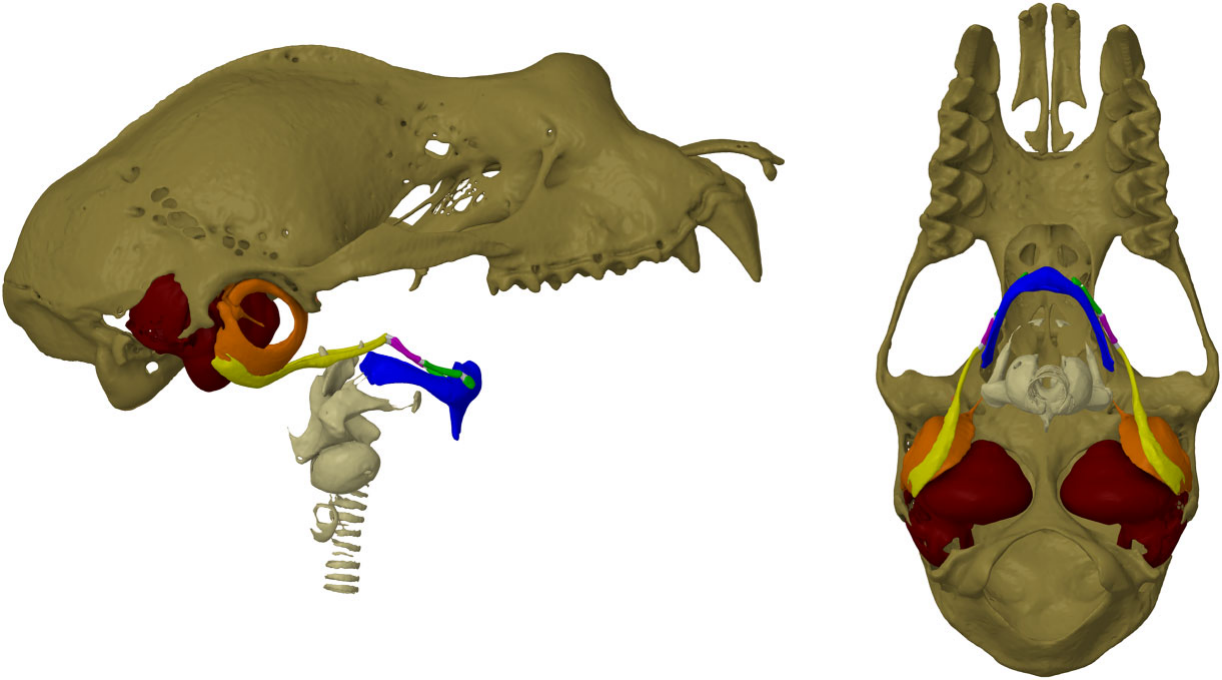
Volume-rendered lateral and ventral views of the cranium (brown), trachea/larynx (gray), and hyoid apparatus from *R. ferrumequinum.* The hyoid apparatus consists of the fused basihyal and thyrohyals (blue), hypohyal (green), ceratohyal (purple), and stylohyal (yellow) bone(s). The hypohyal, ceratohyal, and stylohyal bones are collectively referred to as the anterior cornu, and the bony segments are connected via cartilaginous joints (gray). In laryngeal echolocators, the stylohyal bones articulate with the auditory bullae (orange), which houses the TM and middle ear bones that transfer airborne sound to the cochleae (red).

The echolocation calls of bats are generated either via tongue clicking or by vibration of the vocal cords in the larynx. Laryngeal echolocation in bats coincides with adaptations of the skull and neck not found in tongue clicking bats. These adaptations include enlarged basal turns of the cochlea and increased stiffness of the basilar membrane, which increases hearing sensitivity to the higher frequencies associated with echolocation calls (Kössl and Vater 1995; Vater and Kössl 2011). Laryngeal echolocators also have enlarged, reinforced cricoid, thyroid, and arytenoid cartilages, and hypertrophied intrinsic musculature that allows for the production of powerful, high-frequency calls (Carter 2020). Perhaps most notable is the flattened, paddle-like cranial end of the stylohyal bones that articulate with the auditory bullae, which is considered a characteristic indicative of laryngeal echolocation in bats (Veselka et al. 2010). This is unusual among mammals, as the hyoid typically does not articulate with other bones but instead is suspended in the throat via ligaments and muscles and serves as a dynamic anchor for the complex musculature associated with chewing, swallowing, and vocalization. Since the stylohyal bone is the distal portion of the hyoid apparatus, the unique stylohyal-auditory bulla articulation in laryngeal echolocators completes a bony connection from the larynx (site of call production) to the auditory bulla (site of echo reception) via the hyoid apparatus (Fig. 1). This is particularly interesting because neurological research on echolocating bats shows that bats must first register their outgoing calls to subsequently register the returning echoes (O'Neill and Suga 1979; Suga et al. 1979). Given the close proximity of the larynx to the ear, coupling the two would theoretically provide a more direct means of transferring the call from the site of production to the inner ear and therefore the brain.

**Fig. 2:**
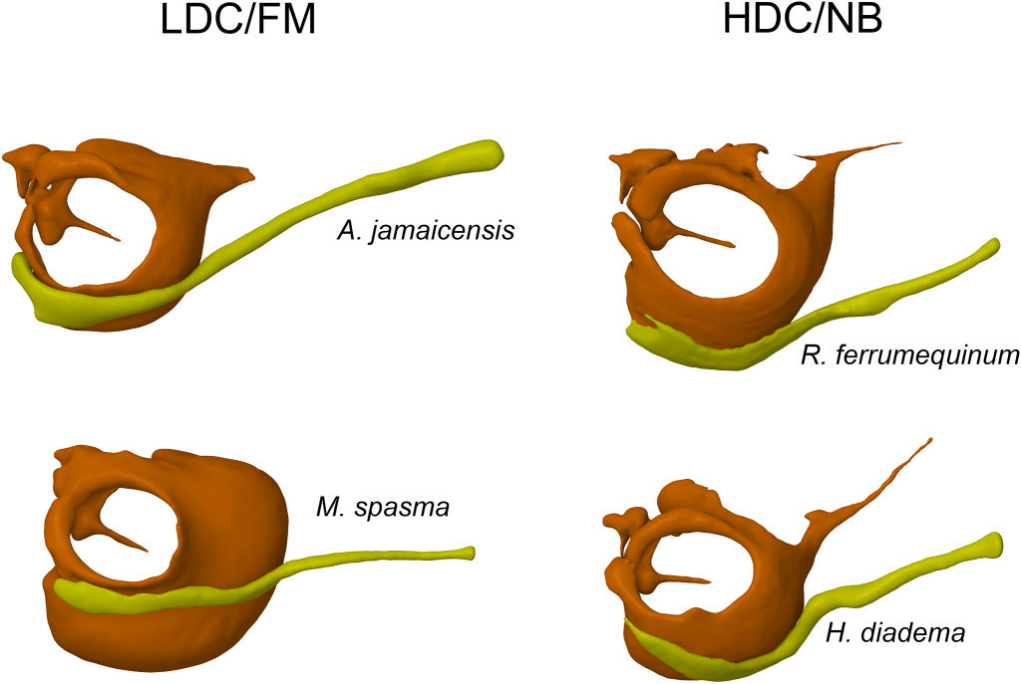
Stylohyal-auditory bulla articulation from LDC/FM and HDC/NB echolocators. The stylohyal (yellow) articulates with the lateral rim of the auditory bulla (orange) in LDC/FM echolocators, whereas the stylohyal articulates with the medial rim of the auditory bulla in HDC/NB echolocators.

Finite-element (FE) modeling of this connection between larynx and ear indicates that sound can be effectively transmitted from the laryngeal surface of the hyoid to the auditory bullae in *Artibeus jamaicensis* (a low duty cycle [LDC]/frequency modulated [FM] echolocator) and *Rhinolophus pusillus* (a high duty cycle [HDC]/narrow band [NB] echolocator) (Snipes and Carter 2022). Here, duty cycle refers to the length of time in a call sequence in which there is out going sound, where LDC bats use 10% of the call sequence and HDC bats use more than 50% of each sequence (Fenton et al. 2012). While both *A. jamaicensis* and *R. pusillus* exhibit spatulate stylohyals that wrap around the bullae, there is variation in the placement and extent to which they articulate with the bulla. The LDC echolocator (*A. jamaicensis*) has stylohyals that wrap around the lateral side of the bullae, while the HDC echolocator (*R. pusillus*) has stylohyals that wrap around the medial rim of the bullae (Fig. 2). Snipes and Carter (2022) did not include a tympanic membrane (TM) in their FE models but instead used the vibration of the bulla as evidence that sound likely moved into the inner ear via bone conduction or a rocking motion of the bulla/TM unit in the lateral-medial plane, which presumably sets the ear ossicles into motion. In that study, we modeled varying levels of constraint (0, 1, 3, and 5 fixed points) on the basihyal to evaluate the effect of muscle attachments and found differences in the performance of our *R. pusillus* and *A. jamaicensis* models. As basihyal constraint was increased, the displacement of the bulla in the *A. jamaicensis* model quickly dropped below the assigned threshold of 2.9e-11 m; and conversely, the bulla of the *R. pusillus* model exhibited displacement peaks above the assigned threshold at all levels of constraint (Snipes and Carter 2022). These results lead us to consider whether HDC echolocators could use vibration of the bulla and bone conduction to transfer sound into the inner ear. The relatively poor performance of the constrained *A. jamaicensis* model (Snipes and Carter 2022), and the placement of the spatulate end of the stylohyal on the bulla (Fig. 2) may mean that excitation of the TM (similar to what airborne sound would do) is the most efficient route for hyoid-borne sound to reach the inner ear in LDC bats.

As the mammalian middle ear is efficient at sound transfer, the connection between the hyoid and ear ossicles may be the most direct way to register outgoing calls to the cochlea. Bats may also use the muscles of the middle ear to attenuate the loud outgoing call so that the soft returning echo can be perceived by the cochlea (Henson 1970). Therefore, a route through the middle ear would enable bats to control the amplitude of the outgoing call arriving at the cochlea. In the present study, we used FE models to assess whether hyoid-borne sound could displace the TM within a range that bats can perceive. To do this, we used experimental data from TM excitation (Manley et al. 1972) and neurophysiological (Heffner et al. 2013) studies of hearing in bats to verify our models and estimate a minimum hearing threshold for TM displacement. We hypothesized that bone-conducted sound through the hyoid would stimulate the TM within a range likely heard by bats. This hypothesis would be supported if displacement of the TM in response to hyoid-borne sound exceeds the estimated hearing threshold. We also hypothesized that the position of the spatulate end of the stylohyal, relative to the plane of the TM, would affect the degree to which the TM is displaced in response to hyoid-borne sound. Support for this would be found if hyoid-borne sound in LDC bats displaced the TM more than in HDC bats (Fig. 2).

## Methods

### Specimens, scanning, and construction of 3D models

**Fig. 3:**
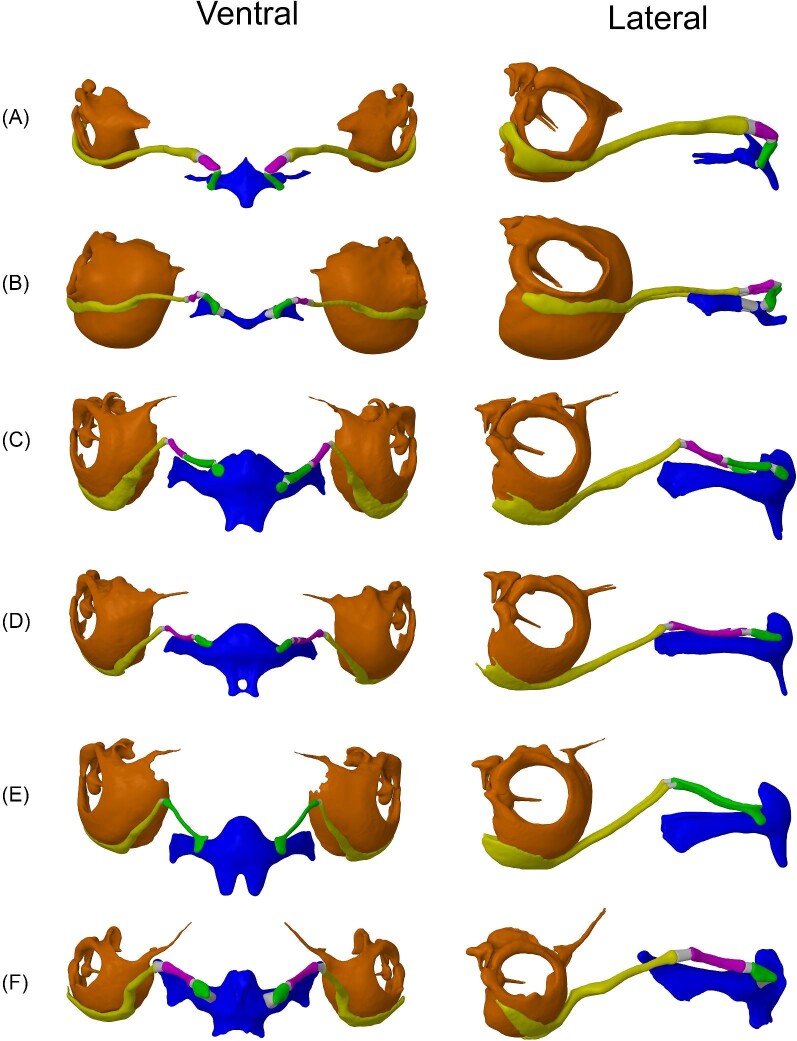
3D models/geometry used for the FE models. Ventral and lateral views of the hyoid apparatus and auditory bullae from (**A**) *A. jamaicensis*, (**B**) *M. spasma*, (**C**) *R. ferrumequinum*, (**D**) *R. rouxi*, (**E**) *R. hildebrandtii*, and (**F**) *H. diadema.* Bones are color coded as follows: fused basihyal and thyrohyals (blue), hypohyal (green), ceratohyal (purple), stylohyal (yellow), auditory bulla (orange), and intervening cartilaginous segments (gray).

Models of the hyoid apparatus, auditory bullae, and TM were built from μCT data of *R. ferrumequinum* (HDC/NB), *R. rouxi* (HDC/NB), *R. hildebrandtii* (HDC/NB), *Hipposideros diadema* (HDC/NB), *Megaderma spasma* (LDC/FM) and *A. jamaicensis* (LDC/FM) specimens (Table 1; Fig. 3A–F). Species were selected to provide variable hyoid morphology for our models, variable phylogenetic position (Yinterochiroptera and Yangochiroptera), and variable call structure at the level of the larynx (e.g., LDC/FM vs HDC/NB). However, we were restricted to species that have extensively ossified hyoids, as this allowed easy segmentation with traditional μCT and did not require staining museum specimens with contrast. Three *Rhinolophus* species were selected as we noticed variation in the morphology of the anterior cornua among species within this genus during our survey of available μCT datasets. Specifically, the number of ossified elements proximal to the basihyal varied in number (Fig. 3C–E), and we wanted to capture potential performance differences resulting from these morphologies. The other families in this study exhibited relatively uniform hyoid morphology within their respective families. Including species that make use of LDC/FM and HDC/NB echolocation (*Rhinolophus* and *Hipposideros*) was important as these two groups have been shown to undergo different ontogenetic steps in the formation of their stylohyal—tympanic bone articulation that leads to different adult morphology (Fig. 2) and may represent convergent evolution of this morphology (Nojiri et al. 2021). Furthermore, the large size of the cochlea in *Rhinolophus* and *Hipposideros* bats results in an auditory bulla that contacts the cochlea and therefore could transmit sound into the inner ear via bone conduction rather than through the TM and ear ossicles.

**Table 1:** Catalog ID, species, data identifier/link, and scan settings for all modeled specimens.

Catalog #	Species	Identifier	Pixel spacing (μm)	Voltage (kV)	Filter (mm)
AMNH 245591	*R. ferrumequinum*	http://n2t.net/ark:/87602/m4/491720	16	80	none
AMNH 48028	*R. rouxi*	http://n2t.net/ark:/87602/m4/491709	15	80	Al-0.5
MVZ 122932	*R. hildebrandtii*	http://n2t.net/ark:/87602/m4/491705	20	80	Al-0.5
MVZ 112095	*H. diadema*	http://n2t.net/ark:/87602/m4/491624	21	80	Al-0.5
UMMZ 163615	*M. spasma*	https://doi.org/10.17602/M2/M57216	46.92	95	none
L-RC:colonyadult	*A. jamaicensis*	http://n2t.net/ark:/87602/m4/491726	18	110	Al-0.5

The *R. ferrumequinum* and *R. rouxi* specimens were provided by the American Museum of Natural History, and the *H. diadema* specimen was provided by the Berkeley Museum of Vertebrate Zoology. All were scanned with a Bruker Skyscan 1273 and reconstructed with Bruker proprietary software at East Tennessee State University. The *M. spasma* specimen was scanned with a Nikon Metrology XT H 225 ST housed in the Earth and Environmental Sciences Department at the University of Michigan and provided by the Museum of Zoology at the University of Michigan. The *A. jamaicensis* specimen was provided by the East Tennessee State University and came from a captive colony housed at the University of Northern Colorado (see Carter et al. 2014). The *A. jamaicensis* specimen was scanned using a Scanco μCT 50 at the Vanderbilt Center for Small Animal Imaging at Vanderbilt University and reconstructed with the Datosǀx 2 reconstruction software (General Electric Company). To capture the shape of the TM in *A. jamaicensis*, contrast enhanced μCT using phosphomolybdic acid (PMA; Gignac et al. 2016) was used, which allowed for the visualization of soft tissues. Following staining with PMA, the specimen was rescanned with a Bruker Skyscan 1273 at East Tennessee State University. All specimens were stored in 70% ethanol.

**Fig. 4:**
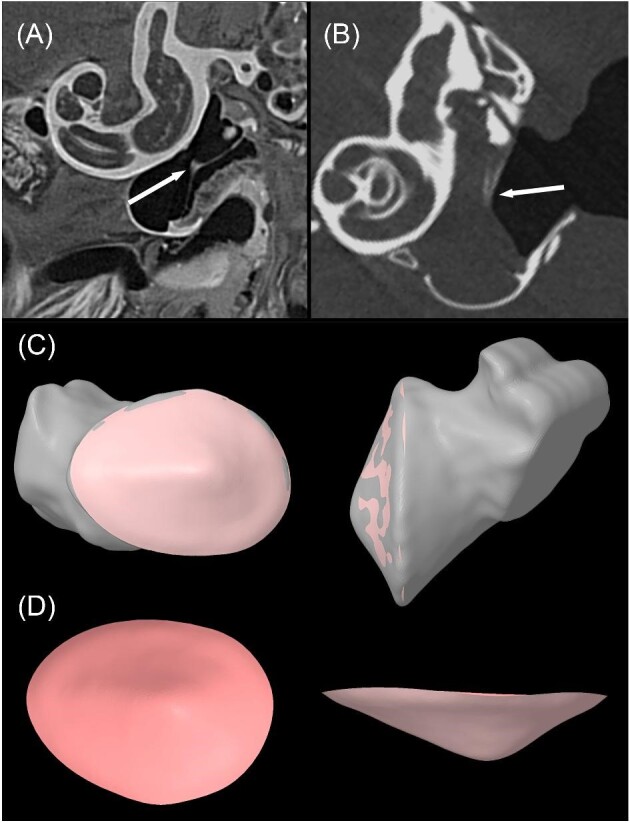
Transverse slices through the auditory bulla and cochlea from a contrast enhanced μCT scan of *A. jamaicensis* (**A**) and a μCT scan of *M. spasma* (**B**). Due to the contrast between the lateral and medial sides of the TM, we were able to segment the negative space on the lateral side (i.e., air) to get the shape of the TM, which was used to construct the TM with NURBS surfaces (**C** and **D**). The medial side of the TM was likely filled with fluid as these specimens were stored in 70% ethanol, resulting in the contrast between the outer and middle ear cavities. Arrows indicate the manubrium of the malleus, which articulates with the medial side of the TM. Note that the slices are from different planes within the middle and inner ears.

Segmentations of the hyoid apparatus (basihyal, hypohyal, ceratohyal, and stylohyal) and the middle ear (auditory bullae, TM, tympanic annulus, and malleus) were created in Dragonfly (Object Research Systems, Montreal, Quebec, Canada) (Fig. 3A–F). The malleus was included in each model due to its attachment to the TM and apparent fusion to the auditory bullae in the scans. For the *A. jamaicensis* model, the contrast enhanced μCT data were imported into the same Dragonfly session as the corresponding traditional μCT scan and aligned using the image registration tool. This allowed for the TM segmentation to be exported and assembled in the anatomically correct location in space relative to the traditional μCT scan. Ultimately, this workflow provided an accurate representation of the TM and annulus for this species and informed us on the precise attachment of the tympanic annulus to the tympanic bone for the remaining models (Fig. 4A). To build the TM for the remaining models, we used the differences in pixel intensity of the space on the medial side of the TM (middle ear) compared to the lateral side (external meatus) (4B). The difference in pixel intensity was likely due to the middle ear of the specimens being filled with 70% ethanol while the meatus was filled with air during scanning. The TM was created by segmenting the air on the lateral side of the TM (acoustic meatus) and segmentations were then exported as triangulated surfaces (.stl files) and assembled in SpaceClaim (Canonsburg, PA, USA). The 3D sketch and skin surface tools were used to wrap a Non-uniform Rational B-spline (NURBS) on the surface of the air segmentation that contacted the TM, creating the surface of the TM for most specimens (Fig. 4C–D). We also laid NURBS on the TM segmented from the contrast enhanced μCT dataset to build the *A. jamaicensis* model. To attach the TM to the bullae, we built the tympanic annulus by laying a NURBS surface on the area where it attaches to the bullae and then used the “funnel” tool to blend that surface to the corresponding edge of the TM. The cartilaginous segments between each bony segment of the hyoid were also created by laying NURBS surfaces on the ends of each corresponding bone, blending them using the “funnel” tool, and then converting them to triangulated surfaces. All triangulated surfaces were then regularized to ensure a uniform mesh, converted to solid bodies (except the TM, which was modeled as a surface body), and saved as Spaceclaim files for FE analysis within ANSYS (Canonsburg, PA, USA).

### FE setup, TM validation, hearing threshold, and harmonic response analyses

**Fig. 5:**
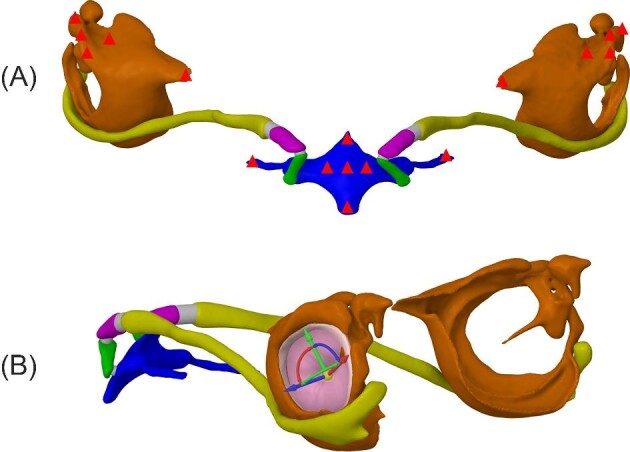
Ventral (**A**) and dorso-lateral (**B**) views of the geometry from the *A. jamaicensis* FE model. All fixed points are indicated with red triangles (**A**) with: four fixed supports on the ventral surface of the basihyal to model muscle attachments, two fixed supports on the ends of the thyrohyals to model their attachment to the larynx, and five fixed supports on the surface of the auditory bullae that closely articulates with the skull. TM displacement data were generated in the axis orthogonal to the plane of the TM, indicated by the blue axis on the triad (**B**). Bones are color coded as follows: fused basihyal and thyrohyals (blue), hypohyal (green), ceratohyal (purple), stylohyal (yellow), auditory bulla (orange), and intervening cartilaginous segments (gray).

We ran a series of harmonic response analyses using modal superposition within ANSYS. Material properties for bone, cartilage, the tympanic annulus, and the TM were assumed to be isotropically elastic (Dumont et al. 2005; Snipes and Carter 2022). Bone, cartilage, the tympanic annulus, and the TM were assigned material properties taken from the literature (Currey 2006; Caminos et al. 2018) (Table 2). Although the TM in bats can range from 20–100 μm thick (Henson 1970), the TM of *A. jamaicensis* was 40 μm thick (contrast-enhanced μCT) and thus used in all TM models for uniformity. This was necessary to ensure all TMs behaved similarly to airborne sound so that differences in its response to hyoid-borne sound could be attributed to hyoid morphology alone. All connections were assigned as bonded (no sliding or separation between faces or edges), and a contact tool was used to ensure contacts had been assigned accurately by ANSYS. Similar to Snipes and Carter (2022), a series of fixed supports were added to the tympanic bullae, basihyals, and thyrohyals to hold the model in space along surfaces that closely articulate with the surrounding anatomy (Fig. 5A). Due to the large number of muscle attachments on the basihyal, we added five fixed supports on its ventral surface of the basihyal which represent attachments of the geniohyoideus, hyoglossus, mandibulo-hyoid, and sterno-hyoideus (Griffiths 1982, 1994; Griffiths et al. 1992). One fixed point was added to the ends of each of the thyrohyals to simulate the articulation with the thyroid cartilage of the larynx. Four fixed points were assigned along the surface of the auditory bullae that closely articulates with the skull. A damping coefficient of 0.02 was applied to the entire model to account for the loss of kinetic/oscillatory energy to the surrounding tissue via friction (Dodge et al. 2012). All solid bodies (bones, cartilages, and the tympanic annulus) were meshed with a fine mesh using quadratic elements, and the TM surface body was meshed with shell elements. The resultant meshes of all six models ranged from 200,000 to 360,000 10-noded tetrahedral elements with 400,000–700,000 nodes. The resultant meshes of the TMs ranged from 4177 to 10,690 shell elements with 8538–19,959 nodes.

**Table 2:** Material properties for bone and cartilage (Currey, 2006) and tympanic membrane (TM) and tympanic annulus (TA) (Caminos et al., 2018) assigned to all FE models.

Material	Density (g/cm^3^)	Young's modulus (Pa)	Poisson's ratio
Bone	2000	2.00E+10	0.3
Cartilage	1100	1.20E+07	0.3
TM	1200	3.20E+07	0.3
TA	1200	3.20E+07	0.3

**Fig. 6:**
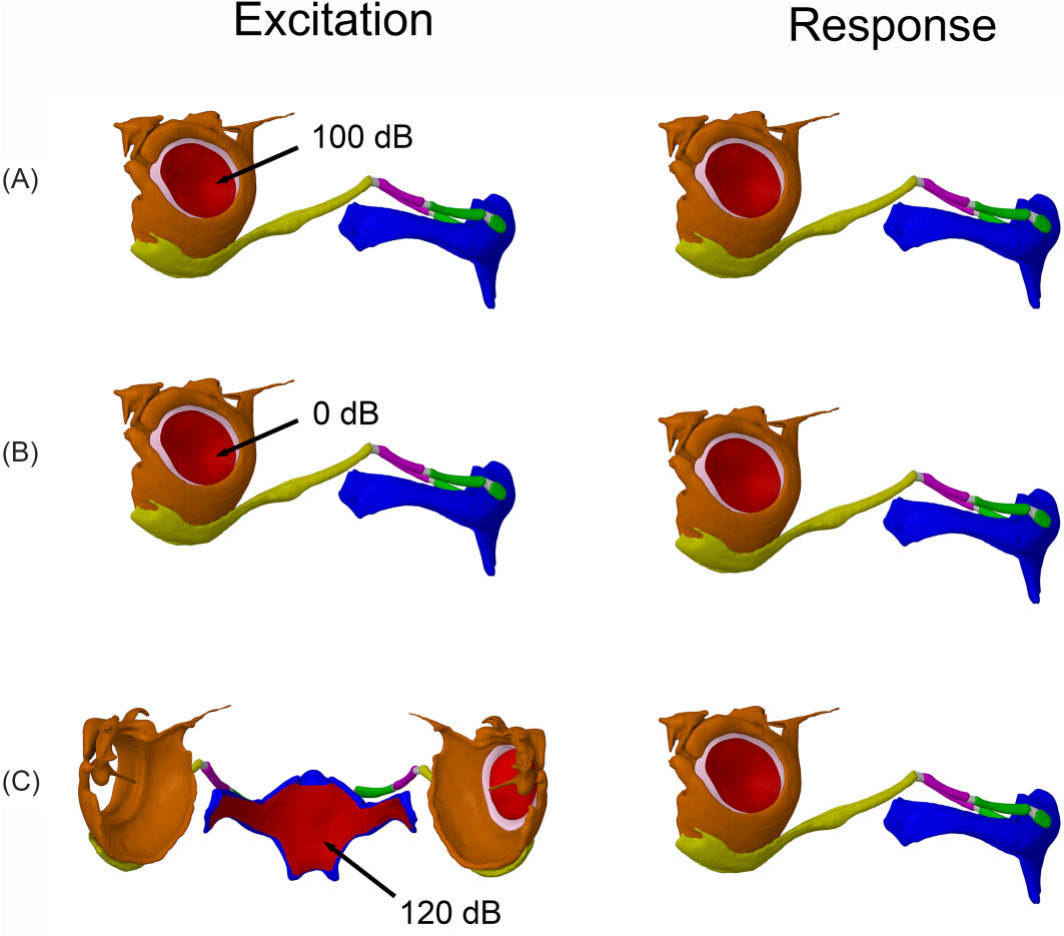
Geometry, including excitation surfaces and surfaces from which results data were generated, for the Harmonic response/modal superposition analyses on *R. ferrumequinum*. (**A**) To verify the TM geometry, the TM was excited with 100 dB sound/pressure on the lateral surface, and response data were generated from the same lateral surface of the TM. (**B**) To establish the displacement hearing threshold, the validated TM was then excited with 0 dB sound/pressure, and response data were generated from the same lateral surface of the TM. (**C**) To mimic an outgoing echolocation call, the laryngeal surface of the basihyal was excited with a 120 dB sound/pressure, and response data were generated from the lateral surface of the TM. Bones are color coded as follows: fused basihyal and thyrohyals (blue), hypohyal (green), ceratohyal (purple), stylohyal (yellow), and auditory bulla (orange). The intervening cartilage segments are gray.

The first analysis was set up to ensure the TM models functioned realistically in response to airborne sound and that the membrane from each model had similar displacement values across frequencies. This was done to ensure that any difference in the TM's response to hyoid-borne sound was due to variation in hyoid morphology and not something intrinsically different between the TM models themselves. To verify the models behaved like that of a real TM, we compared the displacement behavior of the TM models to experimental data collected from the TM of a live bat. Manley et al. (1972) excited the TM of a live *Eptesicus pumilis* with a 100 dB airborne sound and, along with velocity data, recorded an average maximum displacement value of 6.5e–8 m at 2.5 kHz and 2.9e–10 m at 100 kHz with an overall decrease in the average maximum displacement values as frequency increased. To recreate this experiment with the digital models, we excited the lateral side of each TM model with a sinusoidal (harmonic) pressure of 2 Pa (equivalent to 100 dB, SPL ref 20 μPa; all reports of sound pressure level hence forth are referenced to 20 μPa) and recorded the average maximum displacement values of the TM in the axis orthogonal to the plane of the TM (Fig. 5B) from 0–150 kHz (Fig. 6A). Additionally, when exposed to sound, a real TM does not displace as a rigid, piston-like unit but instead with spatial patterns (Khanna and Tonndorf 1972, Cheng et al. 2019). Contour plots were generated at 50, 100, and 150 kHz to confirm the modeled TM displacements in response to airborne sound were realistic.

Once each TM model was validated, we estimated a hearing threshold by measuring the displacement of each TM model when excited with the lowest intensity of airborne sound that is audible in bats. As these are linear models, we divided the TM displacement at 100 dB SPL by a factor of 1e5, which is equivalent to exciting the TM with 20 μPa (0 dB) and measuring the average maximum displacement of the TM from 0–150 kHz (Fig. 6B). We chose 20 μPa (0 dB) because species such as *Desmodus rotundus* and *R. ferrumequinum* have been reported to hear sounds as low as –5 dB and other species have thresholds slightly above 0 dB (Heffner et al. 2013). Although hearing thresholds do vary across species, we feel that 0 dB effectively approximates the lowest hearing threshold in most echolocating bats.

Lastly, to measure the TM response to an outgoing echolocation call, the laryngeal surface of the basihyal was excited with a sinusoidal pressure of 20 Pa (120 dB), and the average maximum displacement values in the axis orthogonal to the plane of the TM were measured from 0–150 kHz (Fig. 6C). The intensity of the outgoing call was chosen based on evidence that *Rhinolophus* bats emit calls as loud as 28.2 Pa (123 dB) measured around 10 cm from the face (Waters and Jones 1995), and phyllostomid fruit bats, like *A. jamaicensis*, can emit calls as loud as 6.3 Pa (110 dB) (Brinkløv et al. 2009). Average maximum displacement values were compared to the estimated hearing threshold to determine if a bat could hear hyoid conducted sound via the TM during call emission. For these models, data points falling above the average hearing threshold (established in the previous analysis) were considered audible, and conversely, data points falling below that threshold were considered inaudible.

## Results

### TM validation through airborne sound and estimated hearing threshold

**Fig. 7:**
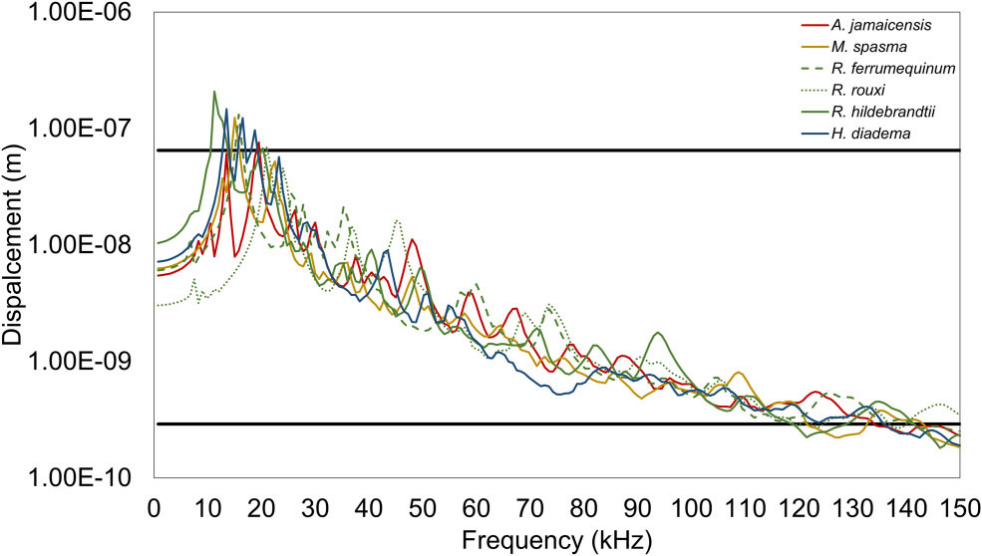
TM displacements (m) in response to a 100 dB excitation on the lateral surface of the TM from *H. diadema* (solid blue line), *R. ferrumequinum* (dashed green line), *R. rouxi* (dotted green line), *R. hildebrandtii* (solid green line), *M. spasma* (solid yellow line), and *A. jamaicensis* (solid red line). Data were generated in the axis orthogonal to the plane of the TM and therefore in the direction that would set the ear ossicles into motion during airborne hearing. The experimental data from Manley et al. (1972) used to verify our TM models are indicated by the black horizontal lines at 6.5e–8 m (2.5 kHz) and 2.9e–10 m (100 kHz).

**Fig. 8:**
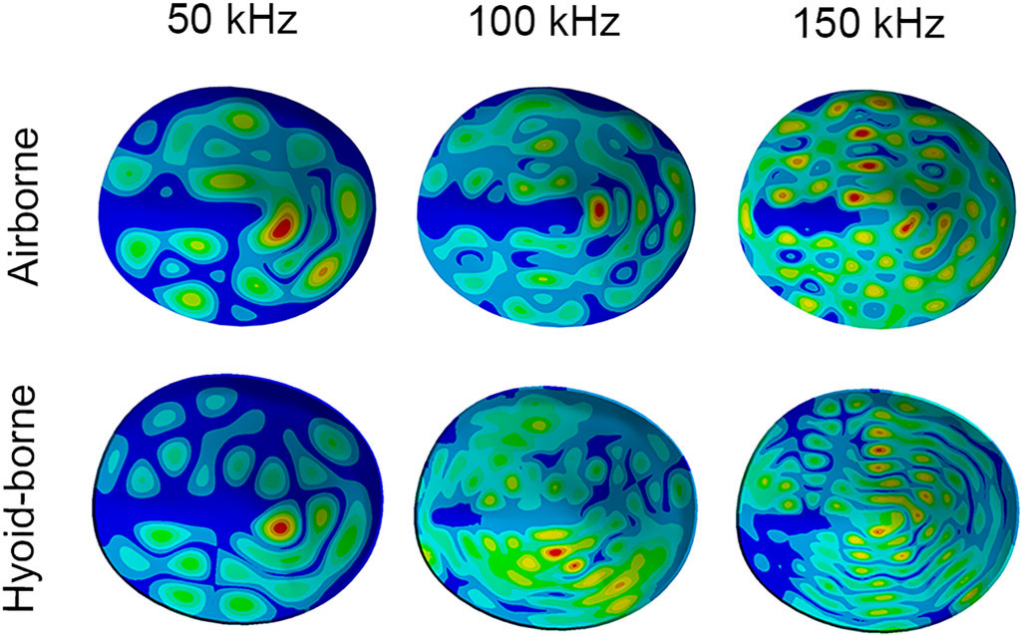
Displacement contour plots of the TM at 50, 100, and 150 kHz in response to airborne and hyoid-borne sound excitations. Warmer colors indicate areas of greater displacement (peaks), while cooler colors indicate areas with less displacement. TM displacements across a range of frequencies are characterized by fewer peaks at lower frequencies and an increase in the number of peaks as frequency increases. Our results show that this is the case in both air- and hyoid-borne excitation.

Although our TM displacement data did not exactly match those reported by Manley et al. (1972), we considered them realistic enough to test our hypotheses (Fig. 7). Additionally, examination of the contour plots at 50, 100, and 150 kHz indicates the TM responds with spatial patterns that are qualitatively similar to those of other species (Fig. 8) (Khanna and Tonndorf 1972; Cheng et al. 2019). Given the similar displacement values of each TM in response to a stimulus at 100 and 0 dB (determined in the previous analysis) across all frequencies (0–150 kHz), these data were averaged and used to represent the estimated hearing threshold across all models in this study.

### Vibroacoustic response of the TM to hyoid-borne sound

**Fig. 9:**
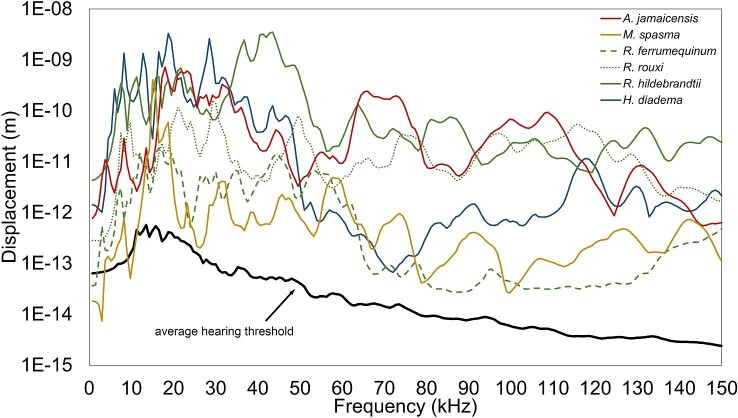
Average maximum TM displacements (m) from 0 to 150 kHz in response to 120 dB excitation on the laryngeal surface of the basihyal from *H. diadema* (solid blue line), *R. ferrumequinum* (dashed green line), *R. rouxi* (dotted green line), *R. hildebrandtii* (solid green line), *M. spasma* (solid yellow line), and *A. jamaicensis* (solid red line). Data were generated in the plane orthogonal to the plane of the TM and therefore in the direction that would set the ear ossicles into motion during airborne hearing. The average maximum TM displacement in response to a 0 dB excitation on the lateral side of the TM was measured from 0 to 150 kHz, and the average from each model was used to represent the average lowest hearing displacement threshold across species (solid black line).

For all species, TM displacements were orders of magnitude greater than the estimated hearing threshold during hyoid-borne propagation of sound (Fig. 9). There were no discernible patterns in the performance of LDC/FM vs HDC/NB models in displacing the TM, nor were there obvious effects due to the variation in morphology of the proximal elements of the anterior cornua in the different *Rhinolophus* species. The contour plots of the TM at 50, 100, and 150 kHz depict spatial displacement patterns similar to that of airborne sound and thus indicate that the TM responds to hyoid-borne sound (Fig. 8).

## Discussion

Our results support the hypothesis that hyoid-borne sound generated during echolocation call emission would stimulate the TM within a range likely heard by bats. Moreover, the TM displacements were orders of magnitude greater than the estimated hearing threshold in most of our models. While our previous work indicated that sound could arrive at the bulla with an intensity that bats could likely hear, we did not test whether it was transferred to the inner ear (or cochlea) through the middle ear or by direct stimulation via bone/soft tissue conduction (Snipes and Carter 2022). The data from the present study show that TM vibration, like that during airborne hearing, can be used to transfer the hyoid-borne call through the middle ear.

Our second hypothesis, that gross differences in stylohyal morphology and its articulation with the bulla would affect the performance of the system, was not supported. The TMs from LDC and HDC bats responded similarly at frequencies below 20 kHz, and although there was some variation at frequencies above 20 kHz, it did not fall along any obvious morphological or phylogenetic lines (Fig. 8). The *R. ferrmequinum* model differed from those of *R. rouxi* and *R. hildebrandtii*, particularly at frequencies above 70 kHz, where there was a reduction in the amplitude of displacements. Compared to the *R. rouxi* and *R. hildebrandtii* models, *R. ferrumequinum* had the largest number of bony elements (four) that make up the anterior cornua which could be responsible for the lower displacements in the higher frequency range. However, if element number of the anterior cornua alone explained variation in the TMs response to hyoid-borne sound, then the *R. rouxi* model (two elements) would have resulted in the largest displacements among *Rhinolophus* models. This was not the case, as the *R. hildebrandtii* model (three elements) had the highest TM displacements across most frequencies. This indicates that the vibroacoustic response of the hyoid, bulla, and TM during sound transfer is complex and hard to estimate using gross morphology alone.

It also means that detailed performance is hard to accurately model without more data on the exact nature of materials, connections, and boundary conditions for each species used. As we assumed many variables to be equal across our models, we included values that do not exactly match reality. For example, we modeled all our TMs using the same material properties and thickness when there is likely variation across species. This is not a problem when testing hypotheses on the overall ability of the system to transmit sound into the ear but can become problematic when testing hypotheses on finer scale performance differences between different groups of bats.

The emitted echolocation pulse can be as high as five orders of magnitude louder than the returning echo, and raises the question of how bats can hear an echo after such a loud initial pulse. Some research suggests that the middle ear muscles contract during vocalization and might attenuate the initial outgoing pulse as it passes through the middle ear (Henson 1965), but experiments on the role of these muscles in attenuating the bats own vocalization have yielded contradictory results and remain unresolved (Neuweiler 2000). If it is the case that middle ear muscles (e.g., stapedius) help attenuate these calls during production, then a route from the hyoid into the TM and through the middle ear would provide a route where attenuation of the call can be modulated as needed. A bone conducted route, where the call passes into the bulla from the hyoid and then stimulates the cochlea directly through vibration, would bypass the ear ossicles and thus not provide an opportunity for attenuation via middle ear muscles. Of course, some bats could use a combination of the two routes (middle ear and bone conducted) to stimulate the cochlea and provide the neurologic registration of the outgoing call in the brain. This scenario is more likely in *Rhinolophus* and *Hipposideros*, as they possess large cochlea that contact the bulla and could therefore transfer a vibration from the bulla directly into the cochlea. Furthermore, our previous models found that *R. pusillus* was capable of effectively displacing the bulla during hyoid-borne sound transfer, whereas *A. jamaicensis* was not (Snipes and Carter 2022).

As previously mentioned, HDC/NB echolocators would likely experience an outgoing call reaching the TM through the hyoid and a returning echo reaching the TM through the air at the same time. This scenario would result in mixing of the two signals at the TM or ear ossicles. If these two signals are at slightly different frequencies due to Doppler shift, they will presumably create a beat-note through periods of constructive and destructive interference when mixed (Wittrock 2010). If the two arriving calls are at the same frequency and in phase, then TM or ear ossicle displacement is expected to be maximized through constructive interference. Conversely, if the arriving calls are the same frequency but out of phase, then TM or ear ossicle displacement is expected to be attenuated through destructive interference. All of these call interactions require explicit dynamic FE modeling where each time step is defined, and therefore where not modeled in the present study.

While we did include variable hyoid morphology from various bat taxa in this study, the hyoid models all contained a series of bones and cartilaginous segments, which is not the case in all echolocators (Sprague 1943). In some genera of laryngeal echolocators (*Eptesicus, Minipterus*, and *Kerivoula*) the proximal hypohyal is fascial, which may affect the ability of the hyoid to transmit the outgoing call from the larynx to the ear. Interestingly, tongue clicking echolocators in the genus *Rousettus* lack a stylohyal—tympanic bone articulation but do possess a facial attachment between the two bones. If fascial connections within the hyoid pose no problem in transmitting the outgoing call to the ear, then a tongue—generated call could also pass through the hyoid to the ear as the basihyal provides attachment for tongue musculature. Of course, all these hypotheses remain to be tested and warrant further modeling.

In summary, the hyoid of laryngeally echolocating bats can transfer laryngeally produced sound to the ear, but we found no gross morphological patterns associated with sound transfer efficiency. This suggests that hyoid morphology and complexity within and between HDC/NB and LDC/FM echolocators have been minimally affected by selection for sound conduction. Given the range of functions associated with the hyoid, along with specialized vocalizations and call emission in echolocating bats (i.e., nasal vs oral emission), we suggest that a comparative functional study of its mechanical properties alongside a broad morphological evaluation could provide insight into the evolution of echolocation in bats and a novel view of the integration and evolvability of the hyoid apparatus across taxa.

## Data Availability

All triangulated surface models and the μCT datasets from which they were derived are available via a unique identifier on Morphosource (www.morphosource.org) (Table 1).
